# Glioblastoma microenvironment contains multiple hormonal and non-hormonal growth-stimulating factors

**DOI:** 10.1186/s12987-022-00333-z

**Published:** 2022-06-04

**Authors:** Daniel Dahlberg, Jutta Rummel, Sonia Distante, Gustavo Antonio De Souza, Maria Ekman Stensland, Espen Mariussen, Helge Rootwelt, Øyvind Voie, Bjørnar Hassel

**Affiliations:** 1 Department of Neurosurgery, Oslo University Hospital, Nydalen, PO box 4950, 0424 Oslo, Norway; 2 Department of Neurohabilitation and Complex Neurology, Oslo University Hospital, Oslo, Norway; 3 Department of Medical Biochemistry, Oslo University Hospital, Oslo, Norway; 4 Institute of Immunology and Centre for Immune Regulation, Oslo University Hospital, Oslo, Norway; 5 Department of Biochemistry, Universidade Federal Do Rio Grande Do Norte, Natal, RN Brazil; 6 Norwegian Defence Research Establishment (FFI), Kjeller, Norway; 7 Department of Air Quality and Noise, Norwegian Institute of Public Health, Oslo, Norway; 8 Institute of Clinical Medicine, Faculty of Medicine, University of Oslo, Oslo, Norway

**Keywords:** Glioblastoma, Cyst fluid, Growth factors, Erythropoietin, Insulin-like growth factor, Testosterone

## Abstract

**Background:**

The growth of malignant tumors is influenced by their microenvironment. Glioblastoma, an aggressive primary brain tumor, may have cysts containing fluid that represents the tumor microenvironment. The aim of this study was to investigate whether the cyst fluid of cystic glioblastomas contains growth-stimulating factors. Identification of such growth factors may pave the way for the development of targeted anti-glioblastoma therapies.

**Methods:**

We performed hormone analysis of cyst fluid from 25 cystic glioblastomas and proteomics analysis of cyst fluid from another 12 cystic glioblastomas.

**Results:**

Glioblastoma cyst fluid contained hormones within wide concentration ranges: Insulin-like growth factor 1 (0–13.7 nmol/L), insulin (1.4–133 pmol/L), erythropoietin (4.7–402 IU/L), growth hormone (0–0.93 µg/L), testosterone (0.2–10.1 nmol/L), estradiol (0–1.0 nmol/L), triiodothyronine (1.0–11.5). Tumor volume correlated with cyst fluid concentrations of growth hormone and testosterone. Survival correlated inversely with cyst fluid concentration of erythropoietin. Several hormones were present at concentrations that have been shown to stimulate glioblastoma growth in vitro. Concentrations of erythropoietin and estradiol (in men) were higher in cyst fluid than in serum, suggesting formation by tumor or brain tissue. Quantitatively, glioblastoma cyst fluid was dominated by serum proteins, illustrating blood–brain barrier leakage. Proteomics identified several proteins that stimulate tumor cell proliferation and invasiveness, others that inhibit apoptosis or mediate adaption to hypoxia and some that induce neovascularization or blood–brain barrier leakage.

**Conclusion:**

The microenvironment of glioblastomas is rich in growth-stimulating factors that may originate from the circulation, the tumor, or the brain. The wide variation in cyst fluid hormone concentrations may differentially influence tumor growth.

**Supplementary Information:**

The online version contains supplementary material available at 10.1186/s12987-022-00333-z.

## Introduction

Glioblastoma, an especially aggressive form of primary brain tumor, develops due to gene mutations [2]. Tumor growth, however, is influenced by factors in the tumor microenvironment, notably the extracellular fluid [13, 45, 51]. Obtaining extracellular fluid from solid tumors in human patients may be challenging, but 5–20% of glioblastomas form fluid-filled cysts that are lined with tumor tissue [4, 27, 53]. The formation of these cysts, which are different from necrotic tumor tissue, probably relies on leakage of plasma proteins into the tumor environment due to dysfunction of the blood–brain barrier (BBB), leading to an increased tissue oncotic pressure that draws water into the tumor [2, 36, 56]. The cyst fluid, which may be obtained during tumor surgery, reflects the tumor’s extracellular fluid phase. Glioblastoma cyst fluid stimulates the growth of cultured glioma cells and astrocytes [40, 62], suggesting the presence of growth factors. One growth factor that has been identified in glioblastoma cyst fluid is vascular endothelial growth factor (VEGF,[57]. Anti-VEGF therapy is a treatment option for glioblastoma, but has not been highly successful [26], possibly reflecting the presence of growth factors other than VEGF in the glioblastoma microenvironment.

The purpose of this study was to see what growth factors glioblastoma cells might be exposed to in their microenvironment. Identification of tumor growth factors may pave the way for the development of new diagnostic methods and targeted anti-glioblastoma therapies. Therefore, we performed hormone and proteomics analyses of cyst fluid from patients with cystic glioblastomas.

## Materials and methods

### Patients and Samples

The study was approved by the Regional Committees for Medical and Health Research Ethics of Norway (Approval #2012/718) and was conducted in accordance with the Declaration of Helsinki. All participants gave informed, written consent.

Patients with cystic glioblastomas were recruited at the Department of Neurosurgery, Oslo University Hospital, 2012–2020. Patients underwent magnetic resonance imaging (MRI) investigation at 1.5 Tesla. The following MRI modalities were available for all patients: T1-weighted images obtained before and after the administration of intravenous gadolinium-based contrast agent, T2-weighted images, and T2-weighted fluid attenuated inversion recovery (FLAIR) images. During tumor surgery, which took place between 10 a.m. and 4 p.m., the cyst fluid volume, which corresponded closely to the volume measured by MRI (see below), was harvested in Vacuette blood collection tubes without additives (Greiner Bio-One, Kremsmünster, Austria). Samples were rapidly centrifuged at 3000*g* for 10 min at 4 °C, and the supernatants (1 mL) were stored in aliquots at − 70 °C in Axigen 1.8 mL microtubes (Corning, Reynosa, Mexico) until analysis. In 15 patients, parallel blood samples were drawn during tumor surgery for serum analysis.

The following parameters were recorded from the MRIs: tumor volume, cyst volume, peritumoral edema, tumor location, cyst location (eccentric versus central), non-contrast enhancing tumor, and whether the tumor was multifocal or not. Tumor size, cyst volumes and peri-tumoral edema were measured in the MR images by two of the authors (JR and DD), who were blind to the protein or hormone profiles of the patients. Segmentation of MR images was done semi-automatically with Smartbrush (Brainlab, Feldkirchen, Germany) in the group of patients that underwent hormonal analysis or manually with Iplan Cranial Planning Software (Brainlab) in the group of patients that underwent proteomics analysis. Peritumoral edema was classified as none (0), mild (1), or moderate/severe (2) as described [50]. In addition, we registered the maximum distance of the edema (from the outer margin of contrast enhancing tumor to the outer edge of edema). Glioblastomas were characterized genetically with respect to isocitrate dehydrogenase (IDH) mutations as part of hospital routine.

### Analysis of hormones and serum proteins in cyst fluid

Cyst fluid from 25 cystic glioblastomas was analyzed with respect to growth hormone, insulin, triiodothyronine, estradiol, progesterone, serum albumin, apolipoprotein A-I, electrolytes and glucose. These analyses were performed as per hospital routine on a Cobas platform (F. Hoffman-La Roche Ltd., Basel, Switzerland) according to the manufacturer’s instructions. Insulin-like growth factor 1 (IGF-1), erythropoietin, and sex hormone-binding globulin (SHBG) were analyzed by an immunoluminometric assay (Immulite 2000xpi; Siemens Healthineers, Erlangen, Germany). Testosterone was analyzed by liquid chromatography-tandem mass spectrometry (LC–MS/MS, 1290 UHPLC system and 6490 tandem mass spectrometer from Agilent, Santa Clara, CA, USA). Serum samples (n = 15) were analyzed with respect to hormones, serum proteins, and electrolytes with the above methods. All cyst fluid and serum concentrations of hormones and serum proteins were within the methods’ ranges and coefficients of variation were within accredited ranges.

### Proteomics analysis

Cyst fluid from a separate cohort of 12 patients with glioblastoma underwent proteomics analysis. Cyst fluid supernatants (see above) were treated as described [18]. Briefly, 10 μL sample fluid were mixed with 200 μL of 0.1% ProteaseMax buffer (Promega, Madison, WI, USA) dissolved in ammonium bicarbonate, 50 mmol/L, pH 7.8. A volume corresponding to 100 μg of protein was in-solution digested in 0.2% ProteaseMax according to manufacturer instructions, using porcine trypsin (MS-grade, sequence-modified, Promega), and samples were incubated at 37 °C overnight. ProteaseMax was degraded by adding trifluoroacetic acid to a final concentration of 0.5% (vol/vol). Samples were left at room temperature for 5 min before precipitates were removed by centrifugation at 14000*g* for 10 min. Before LC–MS injection, the sample peptides were purified on C18 microcolumns (Empore Extraction Disk, Varian, St. Paul, MN, USA).

Protein separation and identification were done on an Easy nLC1000 nano-LC system connected to a quadrupole–Orbitrap (QExactive) MS (ThermoElectron, Bremen, Germany) equipped with a nanoelectrospray ion source (EASY-Spray, Thermo). All MS raw files were submitted to MaxQuant software version 1.5.2.8 for protein identification [3]. Parameters were set as described [18]. We first used a search error window of 20 ppm prior to mass recalibrations of acquired data, followed by a main search error window of 6 ppm. Trypsin without proline restriction enzyme option was used, with two allowed miscleavages. ‘Minimum unique peptides’ was 1; allowed false discovery rate was 1% for peptide spectrum matches and for protein identification.

### Data presentation and statistics

Concentrations of hormones and serum proteins are given as mean ± SD values. In addition, cyst fluid hormone levels are given as full ranges. The data were normally distributed according to the D'Agostino & Pearson normality test. Proteomics data are given as the log-10 transformations of the MS raw data that represent the area under the curve for the individual proteins, mean ± SD values, which were calculated only from samples in which the protein in question could be detected; the number of positive samples is given for each protein in the tables. Differences between groups were analyzed with Student’s two-tailed *t*-test, paired or unpaired, as appropriate. Correlations are given as Pearson’s correlation coefficients, except for correlations between degree of edema (see above; [50]) and cyst fluid levels of hormones and serum proteins, which were evaluated with Spearman’s test. p-values < 0.05 were considered significant.

## Results

### Patient characteristics and tumor details

The 37 glioblastoma patients were 15 women and 22 men, who were 42–79 years old at surgery (median age 59 years). Glioblastoma tumor size was 3–47 cm^3^ (median value 20 cm^3^). Glioblastoma cyst volume was 2–85 cm^3^ (median value 24 cm^3^). Cyst volume correlated with tumor size (r = 0.45; p = 0.0054). Tumors were localized in any of the cerebral lobes: 11 patients had a tumor that primarily resided in a frontal lobe, 8 of them on the right side; 19 patients had a tumor primarily in a temporal lobe, of which 11 were on the right side; 5 patients had a tumor in a parietal lobe, of which 2 were on the right side; 2 patients had a tumor in an occipital lobe, of which one was on the right side. Nine patients had multifocal tumors. The cyst was located centrally in the tumor in 12 patients; in the rest of the patients, the cyst was eccentric/exophytic relative to the main tumor mass, but lined with tumor tissue, as could be verified at surgery. There was no significant difference between patients with central or eccentric cysts with respect to tumor volume (p = 0.71), cyst volume (p = 0.17), extent of peri-tumoral edema (p = 0.45), or survival (p = 0.69).

**Fig. 1:**
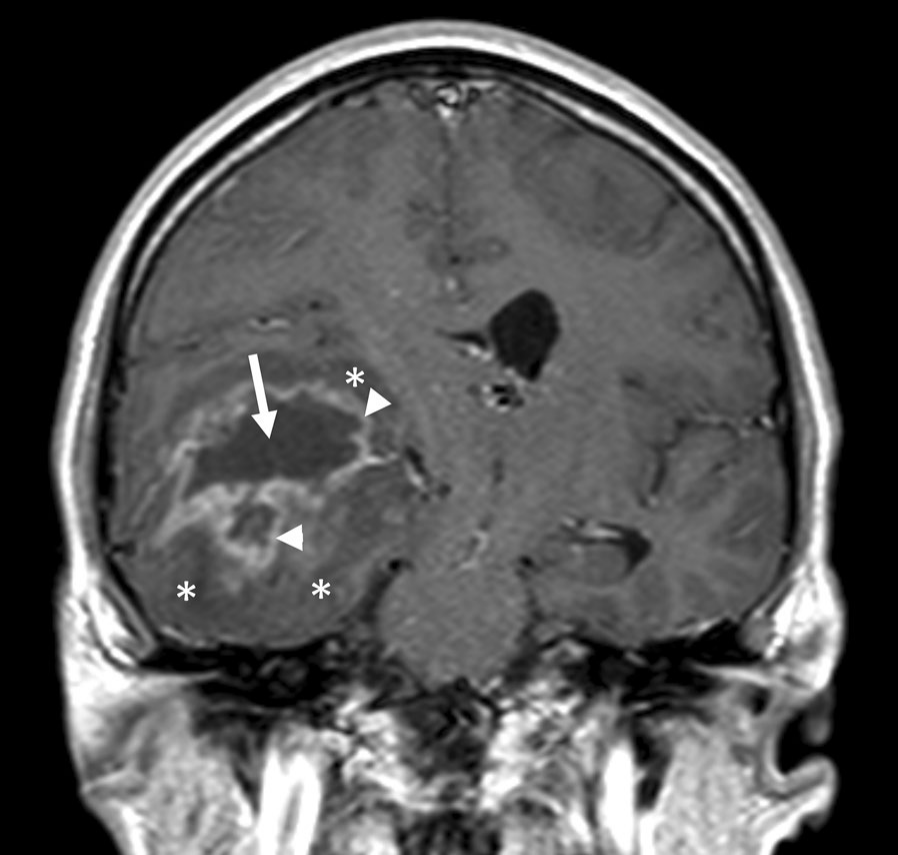
Magnetic resonance image (MRI) of cystic glioblastoma. A T1-weighted MRI obtained after intravenous infusion of a gadolinium-based contrast agent shows a contrast-enhancing cystic glioblastoma in the right temporal lobe. The arrow indicates the cyst. Arrowheads indicate contrast-enhancing tumor, in which leaky capillaries allow the contrast agent to escape into the tumor tissue. Asterisks indicate the peri-tumoral edema, which appears darker than the surrounding brain tissue

Five tumors harbored IDH1 mutations; these were not different from wild type tumors with respect to tumor size (p = 0.55) or cyst volume (p = 0.29). All tumors were contrast-enhancing lesions on MRI, and all but one had a peri-tumoral edema (Fig. 1), both features characteristic of BBB dysfunction [23, 35]. Patients received oral methylprednisolone treatment for 0–30 days prior to neurosurgery to reduce peri-tumoral edema. The initial dose was 16 mg four times per day, which was gradually reduced to 2 mg four times per day, over six days. Thus, some patients received a short, high-dose treatment, others received a more protracted treatment at lower doses, whereas three patients, who underwent emergency surgery, received no methylprednisolone treatment at all. None of the patients received insulin treatment.

### Cyst fluid concentrations of hormones and serum proteins

In cyst fluid from 25 patients with cystic glioblastomas (nine women and 16 men), several hormones were present: IGF-1, insulin, erythropoietin, growth hormone, testosterone, estradiol, and free triiodothyronine (Table 1). Concentrations varied widely between patients. For example, the concentrations of insulin and erythropoietin in glioblastoma cyst fluid varied more than 90-fold, and the concentrations of testosterone and triiodothyronine varied more than tenfold between patients. Testosterone was present at higher concentrations in cyst fluid from male than from female patients (p = 0.0039), while there was no significant difference between male and female patients with respect to estradiol or SHBG levels. Progesterone was not detectable in the cyst fluid of any patient (limit of detection: 1.5 nmol/L).

**Table 1:** Hormones, serum proteins, and electrolytes in cyst fluid from glioblastomas and from serum

	Unit	Glioblastoma cyst fluid level (n = 25)	Serum level (n = 15)	Serum reference values
IGF-1	nmol/L	7.3 ± 3.7^a^ Range: 0–13.7	15.6 ± 4.8	4–22
Insulin	pmol/L	24 ± 31^a^ Range: 1.4–133	105 ± 43	≤ 160
Erythropoietin	IU/L	88 ± 105^a^ Range: 4.7–402	4.1 ± 2.0	4.3–29
Growth hormone	µg/L	0.2 ± 0.2 Range: 0–0.9	0.1 ± 0.1	< 0.5
Testosterone (M)	nmol/L	5.0 ± 2.7 (n = 16)^b^ Range: 0.9–10.1	7.3 ± 3.7 (N = 11)^b^	4.6–24
Testosterone (F)	nmol/L	0.7 ± 0.4 (n = 9) Range: 0.2–1.5	0.5 ± 0.3 (n = 4)	≤ 1.1
Estradiol (M)	nmol/L	0.22 ± 0.28 (n = 16)^a^ Range: 0–1.0	0 (n = 11)	0.06–0.14
Estradiol (F)	nmol/L	0.09 ± 0.10 (n = 9) Range: 0–0.2	0.07 ± 0.10 (n = 4)	< 0.12
Triiodothyronine	pmol/L	3.2 ± 2.1 Range: 1.0–11.5	3.4 ± 0.7	2.8–7.0
SHBG (M)	nmol/L	19 ± 11 (n = 16)	22 ± 11 (n = 11)	15–90
SHBG (F)	nmol/L	26 ± 9 (n = 9)	35 ± 7 (n = 4)	23–100
Albumin	g/L	28 ± 9^a^	40 ± 4	38–52
Apolipoprotein A-I	g/L	0.5 ± 0.3^a^	1.5 ± 0.3	1.0–2.3
Sodium	mmol/L	140 ± 20	139 ± 4	137—145
Potassium	mmol/L	3.8 ± 0.7	4.1 ± 0.5	3.6–4.6
Calcium	mmol/L	1.67 ± 0.47^a^	2.28 ± 0.15	2.20–2.55
Glucose	mmol/L	1.7 ± 1.3^a^	6.2 ± 1.1	4.2–6.2

Cyst fluid from 25 glioblastoma patients was analyzed with respect to hormones, serum proteins, electrolytes and glucose. For 15 glioblastoma patients, parallel serum samples were available. Data are mean ± SD values.

All p-values were obtained with Student’s unpaired 2-tailed *t*-tests, not corrected for multiple comparisons. Serum reference values are from The Oslo University Hospital Hormone laboratory. Testosterone reference values are for men (M) > 41 years and for women (F) > 50 years. Estradiol reference values are for men > 17 years and for women > 50 years. SHBG reference values are for men > 60 years and for women > 18 years. Zero values are below the limits of detection, which were: IGF-1: 1.95 nmol/L, growth hormone: 0.030 µg/L, estradiol: 0.04 nmol/L. Abbreviations: IGF-1: insulin-like growth factor 1; SHBG: sex hormone-binding globulin

^a^significantly different from serum values; p < 0.001

^b^different from corresponding testosterone values in female patients; p < 0.004

Parallel samples of cyst fluid and serum were obtained from 15 glioblastoma patients. The concentration of some hormones in cyst fluid was lower than (IGF-1, insulin) or similar to (growth hormone, testosterone, triiodothyronine) the serum concentration (Table 1). In contrast, the concentration of erythropoietin and (in male patients) estradiol was significantly higher in cyst fluid than in serum. The mean levels of albumin and apolipoprotein A-I in cyst fluid were 64% and 34% of those in serum, respectively, and the variability in concentration was greater in cyst fluid than in serum, as could be seen from the standard deviations. The level of SHBG was not significantly different in cyst fluid and serum. The corresponding cyst fluid:serum ratios are given in Table 2. Expressed as cyst fluid:serum ratio, the value for albumin was not different from 1. Sodium and potassium were similar in cyst fluid and serum (Table 1), but the total concentration of calcium was lower in cyst fluid, probably reflecting the lower level of albumin, which carries calcium. The level of glucose in cyst fluid was < 0.1–4.9 mmol/L, in agreement with a previous study [6], whereas serum glucose was 4.0–8.1 mmol/L. The mean level of glucose in cyst fluid was 27% of the serum level. The three tumors harboring IDH1 mutations in this group of 25 glioblastomas did not stand out in any way with respect to cyst fluid concentrations of hormones, serum proteins, electrolytes, or glucose.

**Table 2:** Cyst fluid:serum ratios for hormones and serum proteins

IGF-1	Insulin	EPO	GH	Testosterone	T3	APOA1	Albumin	SHBG
0.4 ± 0.2	0.2 ± 0.4	24 ± 32	1.9 ± 1.3	0.9 ± 0.7	1.1 ± 0.7	0.4 ± 0.2	0.9 ± 0.3	0.8 ± 0.4

Cyst fluid and serum from 15 glioblastoma patients were analyzed with respect to hormones and serum proteins. Data are mean ± SD values. Estradiol was not detected in several serum samples and a ratio could therefore not be calculated. Abbreviations: APOA1: apolipoprotein A–I; EPO: erythropoietin; GH: growth hormone; IGF-1: insulin-like growth factor 1; SHBG: sex hormone-binding globulin; T3: triiodothyronine

We asked to what extent glioblastoma cyst fluid reflected serum concentrations of hormones. The concentration of testosterone in cyst fluid and serum was significantly correlated (r = 0.68; p = 0.0057; n = 15), whereas the concentrations of IGF-1, insulin, erythropoietin, growth hormone, estradiol, and triiodothyronine in cyst fluid and serum were not significantly correlated (correlation coefficients 0.03–0.42; n = 15).

In the cysts that lay centrally in the tumor, the concentration of SHBG tended to be higher than in those that lay eccentrically: 25 ± 14 vs. 16 ± 9 nmol/L (p = 0.059; n = 9 and 16, respectively). For the hormones and the other plasma proteins there were no significant differences between central and eccentric cysts (p-values > 0.14).

To examine whether a dysfunctional BBB (or blood-tumor barrier) might contribute to the influx of hormones into the glioblastoma cyst, we looked for correlations between cyst fluid levels of serum proteins and hormones (Table 3). The serum proteins apolipoprotein A-I, albumin, and SHBG span molecular weights from 28 to 100 kDa. IGF-1 and growth hormone both appear in isoforms with molecular weights in this range [47]. Cyst fluid levels of IGF-1 and growth hormone correlated with cyst fluid levels of serum proteins, suggesting the importance of BBB dysfunction, whereas levels of erythropoietin and insulin did not correlate with serum protein levels in cyst fluid. The level of testosterone correlated with the level of albumin, which may carry testosterone [5], but not with SHBG levels. The levels of albumin and apolipoprotein A-I correlated closely.

**Table 3:** Correlations between cyst fluid concentrations of serum proteins and hormones

	APOA1	Albumin	SHBG
	(Mw 28)	(Mw 66)	(Mw ~ 100)
IGF-1	0.52^b^	0.34	0.47^a^
Insulin	0.16	− 0.03	0.13
Erythropoietin	− 0.07	− 0.28	− 0.20
Growth hormone	0.57^b^	0.54^b^	0.65^c^
Testosterone	0.20	0.45^a^	0.27
Estradiol	− 0.01	0.22	0.07
Triiodothyronine	0.48^a^	0.30	0.05
SHBG	0.56^b^	0.54^b^	
Albumin	0.97^c^		

Cyst fluid from 25 patients with cystic glioblastomas were analyzed with respect to hormones and serum proteins, and MRIs were used to calculate the volumes of tumors and cysts. Correlations were calculated with Pearson’s test. Asterisks: ^a^p < 0.05; ^b^p < 0.01; ^c^p < 0.001. Abbreviations: APOA1: apolipoprotein A–I; IGF-1: insulin-like growth factor 1; Mw: weight; SHBG: sex hormone-binding globulin

Cyst fluid concentration of insulin correlated with duration of methylprednisolone treatment, whereas the concentration of growth hormone correlated inversely with treatment duration (Table 4). In contrast, the serum concentrations of insulin and growth hormone did not correlate with duration of methylprednisolone treatment. For the other hormones, no significant correlation was seen between length of methylprednisolone treatment on the one hand and cyst fluid or serum concentrations on the other. Duration of methylprednisolone treatment did not correlate with cyst fluid concentrations of apolipoprotein A-I (r = 0.03) or albumin (r = 0.005), but it correlated inversely with SHBG (r = − 0.50; p = 0.011).

**Table 4:** Correlation between duration of corticosteroid treatment and levels of insulin or growth hormone in cyst fluid and serum

		Pearson’s r =	p-value
Insulin	Cyst fluid (n = 25)	+ 0.48	0.015
	Serum (n = 15)	+ 0.11	0.7
Growth hormone	Cyst fluid (n = 25)	− 0.50	0.011
	Serum (n = 15)	− 0.07	0.8

Prior to neurosurgery, 25 patients with cystic glioblastoma received corticosteroid treatment for 0–30 days to reduce peri-tumoral edema. For 15 patients parallel serum samples were available for analysis. Data are Pearson’s correlation coefficients and corresponding p-values

Cyst fluid concentrations of growth hormone, testosterone, SHBG, and albumin correlated with tumor volume (Table 5). The concentration of estradiol correlated sub-significantly (p = 0.082) with tumor volume, but more strongly with cyst volume. The concentration of SHBG also correlated with cyst volume, but testosterone did so only sub-significantly. The concentration of insulin correlated with extent of peri-tumoral edema; this correlation could be influenced by the length or, dosage of corticosteroid treatment (Table 4), which was determined by edema-related symptoms.

**Table 5:** Correlations between glioblastoma cyst fluid hormones and serum proteins and the volumes of tumors and cysts and degree of peri-tumoral edema

	Tumor volume		Cyst volume		Peritumoral edema	
	r	p	r	p	r	p
IGF-1	0.11		0.38	0.061	0.08	
Insulin	− 0.03		− 0.26		0.60	0.0016
Erythropoietin	0.03		− 0.08		− 0.04	
Growth hormone	0.40	0.046	0.26		0.31	
Testosterone	0.42	0.039	0.35	0.086	− 0.04	
Estradiol	0.36	0.086	0.50	0.011	− 0.08	
Triiodothyronine	− 0.18		− 0.14		− 0.27	
SHBG	0.61	0.0014	0.60	0.0014	0.02	
Albumin	0.48	0.015	0.20		0.25	
Apolipoprotein A-I	0.26		0.28		0.02	

Twenty-five cystic glioblastomas were analyzed with respect cyst fluid concentrations of hormones and serum proteins and tumor and cyst volumes and peri-tumoral edema. Data are correlation coefficients (r) and corresponding significant or near-significant p-values (p); Pearson’s test. Abbreviations: IGF-1: insulin-like growth factor 1; SHBG: sex hormone-binding globulin

Survival of the glioblastoma patients after surgery correlated inversely with cyst fluid level of erythropoietin (r = − 0.44; p = 0.045). The cyst fluid concentration of the other hormones and serum proteins did not correlate with survival (r-values − 0.17–0.28, p-values: > 0.22).

### The proteome of glioblastoma cyst fluid

Glioblastoma cyst fluid from a separate cohort of 12 patients underwent proteomics analysis. In each sample, 314–901 different proteins were identified, illustrating the complexity and the variability of the tumor microenvironment; 146 proteins were detected in all 12 glioblastomas (Additional file 1: Table 1). Plasma proteins (e.g., serum albumin, apolipoproteins, alpha-1-antitrypsin, gammaglobulins, complement factors) accounted for 70% of the 146 proteins, reflecting the derangement of normal BBB function in glioblastoma [23, 35]. The remaining 30% of the proteins are also present in plasma [49], but could also originate from tumor or brain cells, e.g. cytoskeleton proteins and intracellular enzymes.

Several proteins that are involved in tumor growth were present in glioblastoma cyst fluid (Table 6; for references, see “Discussion” section): SPARCL1, IGF-BP2, osteopontin, FAM3C, TREM2, CD166, and prosaposin promote tumor cell proliferation, migration, and invasiveness. Kininogen-1/bradykinin stimulates glioma cell migration; the kininogen-activating enzyme kallikrein, was present in all glioblastoma samples.

**Table 6:** Tumor growth-associated proteins in cyst fluid from cystic glioblastomas

Protein	Function in tumor biology	N	Mean ± SD
SPARCL 1	Glioma cell proliferation and invasion	12	9.0 ± 0.4
IGF-BP 2	Glioma cell invasion. Vasculogenic mimicry	12	8.3 ± 0.6
Kininogen-1/bradykinin	Glioma cell migration. Endothelial leakage	12	9.6 ± 0.2
Kallikrein	Endothelial leakage. Anti-apoptotic	12	7.9 ± 0.5
Osteopontin	Tumor cell migration	12	9.9 ± 0.5
FAM3C	Invasion. Proliferation	11	7.9 ± 0.3
TREM2	Glioma cell invasion. Proliferation	9	7.8 ± 0.3
CD166 (secreted)	Glioma cell invasion	9	7.7 ± 0.5
Prosaposin	Invasion. Anti-apoptotic	7	7.5 ± 0.4
Clusterin (secreted)	Anti-apoptotic	12	10.2 ± 0.3
Soluble CD5L	Anti-apoptotic	10	8.1 ± 0.5
HYOU1	Anti-apoptotic. Hypoxia adaptation	8	7.7 ± 0.6
HGFA	Activates HGF	9	7.7 ± 0.4
HRG	Tumor vascularization	12	10.1 ± 0.5
Alpha-2-HS-glycoprotein	Tumor vascularization	12	10.1 ± 0.5
LRG	Tumor vascularization	12	9.9 ± 0.4
GRP78 (secreted)	Tumor vascularization	12	8.6 ± 0.7
MIF	Tumor vascularization	9	7.9 ± 0.6
VEGFA	Tumor vascularization. Vasculogenic mimicry. Endothelial leakage	8	7.7 ± 0.2
HDGF	Tumor vascularization	2	7.8 ± 0.4
Cadherin-5 (soluble)	Endothelial leakage. Vasculogenic mimicry	12	8.2 ± 0.8

Cyst fluid from 12 cystic glioblastomas was sampled during tumor surgery and the proteome was analyzed by tandem mass spectrometry. Column “N” gives the number of samples out of 12 with a positive protein identification. Only samples with a positive protein identification were used to calculate mean ± SD values. Protein levels are given as log-10 of the area under the curve as determined by mass spectrometry. Abbreviations: CD5L: CD5-like protein (CD: cluster of differentiation); FAM3C: family with sequence similarity 3; FGF: fibroblast growth factor; GRP78: glucose-regulated protein 78; HDGF: Hepatoma-derived growth factor; HGF: Hepatocyte growth factor; HGFA: Hepatocyte growth factor activator; HRG: histidine-rich glycoprotein; HYOU1: hypoxia up-regulated protein 1; LRG: Leucine-rich α-2-glycoprotein; MIF: macrophage migration inhibitory factor; SPARCL1: secreted, acidic and rich in cysteine-like 1; TREM2: triggering receptor expressed on myeloid cells 2; VEGFA: vascular endothelial growth factor A

Proteins that have an anti-apoptotic effect on tumor cells or mediate adaptation to hypoxia were detected: clusterin, CD5L, HYOU1, prosaposin, and hepatocyte growth factor activator.

Several proteins that are involved in tumor vascularization were detected: histidine-rich glycoprotein (HRG), α-2-HS-glycoprotein, leucine-rich α-2-glycoprotein (LRG), secreted GRP78, macrophage migration inhibitory factor (MIF), VEGFA, and hepatoma-derived growth factor. Kininogen-1 promotes neovascularization through its product bradykinin.

Identified proteins that are known to participate in inflammatory processes and cause capillary leakage included kininogen-1/bradykinin, kallikrein, HRG, α-2-HS-glycoprotein, LRG, MIF, VEGFA, TREM2, and soluble cadherin-5. However, proteins with presumed anti-inflammatory properties were also detected: apolipoproteins A-I, A-II, and A-IV [10, 14, 44], alpha-1-acid glycoprotein 1 (orosomucoid 1; [33] and alpha-1-acid glycoprotein 2 (orosomucoid 2; [25]. These anti-inflammatory proteins were present in all cyst fluid samples (Additional file 1: Table).

## Discussion

Here we show that glioblastoma cyst fluid, which represents the microenvironment of glioblastoma cells, contains a host of tumor growth factors, hormonal as well as non-hormonal. Growth factor concentrations varied widely between patients: in some samples, growth factors were present at high concentrations, in others, certain growth factors could not be detected.

Some of the hormones in glioblastoma cyst fluid were present at concentrations that have been shown to promote glioblastoma cell growth in culture. In one study, IGF-1 and insulin stimulated the growth of glioblastoma cells at 0.7 nmol/L and 80 pmol/L, respectively [15], which is well within the concentration ranges found in glioblastoma cyst fluid in the present study. Testosterone has been shown to stimulate glioblastoma cell growth at 10 nmol/L [43, 52], a concentration that was within the range in cyst fluid from male patients. In the present study, the cyst fluid concentration of testosterone correlated with tumor volume, possibly illustrating its role in glioblastoma growth. Similarly, the cyst fluid concentration of SHBG and albumin, both of which may carry testosterone [5], correlated with tumor volume. We would like to emphasize, however, that such correlations do not imply causality. One study found estradiol to stimulate glioblastoma cell growth at 10 nmol/L [16], but in none of our samples did we detect estradiol above 1 nmol/L, which could mean that estradiol is of minor importance for glioblastoma growth in vivo. We did, however, see a correlation between cyst fluid estradiol levels and cyst volume, which could suggest a role for estradiol in glioblastoma even at low concentrations. The finding that male patients had a higher estradiol level in cyst fluid than in serum probably reflected aromatase activity in the tumor [21], catalyzing conversion of testosterone to estradiol. The present study confirms a higher exposure of glioblastomas to testosterone in men than in women, but it does not confirm a presumed higher exposure of glioblastomas to estradiol in women [19], as the concentration of estradiol in cyst fluid from male patients was at least as high as the concentration in cyst fluid from female patients.

Available evidence suggests roles for growth hormone [7], erythropoietin [38], and triiodothyronine [34] in glioblastoma growth, but further studies are needed to establish whether the growth-stimulating concentrations of these hormones are within ranges found in the glioblastoma microenvironment. In the present study, the cyst fluid concentration of growth hormone correlated with tumor volume, and the concentration of erythropoietin correlated inversely with survival, possibly illustrating a role for these hormones in the growth of glioblastomas. The presence of IGF-1, insulin, erythropoietin, and growth hormone, four peptide hormones, in glioblastoma cyst fluid suggests that multiple other peptide hormones may be present in tumor cyst fluid. This conclusion points to the need to explore the effects of peptide hormones on glioblastoma growth more broadly.

The proteomics analysis did not yield absolute concentrations of the various proteins in the glioblastoma cyst fluid, and so we cannot establish whether they were present at levels that would be necessary to support tumor growth. However, previous studies suggest that several of the identified proteins are potent stimulators of tumor cell proliferation or invasiveness, including SPARCL1 [12], IGF-BP2 [20], osteopontin [48], FAM3C [59], TREM2 [61], CD166 [28], prosaposin [1], and kininogen-1/bradykinin [42, 54]. Some of the identified proteins have been shown to inhibit apoptosis or cause tumor cells to adapt to hypoxia, thereby promoting tumor cell survival, including clusterin [41], CD5L [31], HYOU1 [55], prosaposin [1], and hepatocyte growth factor activator [9]. Some of the identified proteins have been shown to promote neovascularization and endothelial permeability, thereby securing the supply of nutrients and growth factors to tumor cells: HRG and α-2-HS-glycoprotein [29], LRG [11, 60], secreted GRP78 [32], MIF [17], VEGFA [37], hepatoma-derived growth factor [24], kininogen-1/bradykinin [29, 63], and soluble cadherin-5 [8]. It is noteworthy that glioblastoma cyst fluid contains several proteins that stimulate angiogenesis through different molecular mechanisms. In addition, cadherin-5, VEGF, IGFBP2, and MMP2 (MMP2 was detected in two glioblastoma cyst fluid samples (Additional file 1: Table 1) are involved in the generation of vasculogenic mimicry in glioblastomas [35]. These findings could explain the limited success of anti-angiogenic treatment specifically targeting VEGF in glioblastoma therapy [26].

All of the tumor growth-promoting factors identified in glioblastoma cyst fluid are known to be present in blood, including hormones, kininogen-1, osteopontin, GRP78, SPARCL1, HRG, LRG, α-2-HS-glycoprotein, MIF, VEGFA, kallikrein, IGFBP2, CD166, clusterin, CD5L, and hepatocyte growth factor activator [49]. This consideration points to blood as one important source of growth-promoting proteins in the glioblastoma microenvironment; the entry of these proteins into the glioblastoma microenvironment would be facilitated by a leaky BBB. BBB leakage was indeed suggested by the correlation between certain hormones (IGF-1 and growth hormone) and serum proteins in glioblastoma cyst fluid. However, erythropoietin and estradiol (in men) were present in glioblastoma cyst fluid at higher concentrations than in serum, suggesting formation of these hormones by tumor or brain tissue, which would explain why their levels did not correlate with the levels of serum proteins and which would be in agreement with previous studies showing erythropoietin and estradiol formation by glioblastoma [21, 46]; erythropoietin formation could be triggered by hypoxic conditions. Likewise, IGF-1 [15], IGFBP2 [35], SPARCL1 [22, 30], kininogen-1 [63], osteopontin [48], and GRP78 [32] are known to be secreted by glioblastoma cells. Thus, the growth-stimulating microenvironment of glioblastoma tumor cells is enriched by tumor cells themselves in an autocrine or paracrine fashion. In the present study, we have analyzed cyst fluid from cystic glioblastomas, but because all glioblastomas are characterized by BBB leakage [23, 35], allowing blood-borne growth factors into the glioblastoma microenvironment, the results reported here could be relevant also for non-cystic glioblastomas.

Methylprednisolone treatment prior to surgery may have increased the cyst fluid level of insulin, as suggested by the correlation between length of treatment and cyst fluid insulin concentration. This would agree with previous studies showing that corticosteroids given over several days stimulate insulin secretion [58]. Similarly, methylprednisolone treatment appeared to reduce the cyst fluid level of growth hormone. This would seem to agree with the suppressive effect of corticosteroids on serum levels of growth hormone [39]. However, no correlation was seen between length of corticosteroid treatment and serum levels of insulin or growth hormone. This discrepancy points to the need for further study of the passage of molecules between the circulation and the glioblastoma microenvironment.

## Conclusions

We asked if the microenvironment of glioblastomas, as reflected in the cyst fluid of cystic glioblastomas, contain growth factors that may contribute to the growth of these aggressive tumors. We found that the glioblastoma cyst fluid contained a host of growth factors, both hormonal and non-hormonal. Many of these growth factors have been shown to stimulate growth of cultured tumor cells. In the present study, cyst fluid concentrations of testosterone and growth hormone correlated with tumor size, whereas the concentration of erythropoietin correlated inversely with patient survival. The concentration of growth factors varied greatly among patients, which could differentially influence tumor growth.

## Supplementary Information

**Additional file 1: Table 1.** Proteins identified in cyst fluid from cystic glioblastomas. Cyst fluid was harvested from 12 cystic glioblastomas. Data are number of samples in which the protein was detected, log-10 values of raw data (area under the curve) from the mass spectrometry analysis; mean ± SD as calculated from the samples with positive identification of the protein in question. On the first page, proteins are listed according to abundance and number of positive samples in glioblastoma cyst fluid. On the second page, proteins are listed alphabetically. In the rightmost columns, data on peptide counts are given together with gene names and FASTA headers.

## Data Availability

The data that support the findings of this study are available on request from the corresponding author. The data are not publicly available due to privacy or ethical restrictions.
